# New species of *Delicata* (Molineidae: Anoplostrongylinae) parasite of *Cabassous tatouay* (Desmarest, 1804) from the Atlantic Forest, Rio de Janeiro, Brazil

**DOI:** 10.3389/fvets.2023.1325263

**Published:** 2024-01-08

**Authors:** Raquel de Oliveira Simões, Beatriz Elise de Andrade Silva, Natalie Olifiers, Cecília Bueno, Arnaldo Maldonado Júnior

**Affiliations:** ^1^Departamento de Parasitologia Animal, Universidade Federal Rural do Rio de Janeiro, Seropédica, Brazil; ^2^Laboratório de Biologia e Parasitologia de Mamíferos Silvestres Reservatórios, FIOCRUZ, Rio de Janeiro, Brazil; ^3^Núcleo de Estudos de Vertebrados Silvestres – NEVS, Universidade Veiga de Almeida, Rio de Janeiro, Brazil; ^4^Museu Nacional, Departamento de Vertebrados, Universidade Federal do Rio de Janeiro, Rio de Janeiro, Brazil

**Keywords:** armadillo, biodiversity, Nematoda, road-killed, Xenarthra

## Abstract

A new species of nematode parasite of the genus *Delicata* (Molineidae: Anoplostrongylinae) is described from the small intestine of a road-killed Greater Naked-tailed Armadillo *Cabassous tatouay* (Cingulata: Chlamyphoridae) on the BR-040 highway in Rio de Janeiro state, Brazil. The genus *Delicata* includes 13 species of parasitizing armadillos and anteaters distributed in Brazil, Argentina, and Trinidad and Tobago. The present species is distinguished from almost all species of *Delicata* by the longest length of the body, except for *D. khalili* and *D. appendiculata.* However, these can be distinguished from each other by the length of the spicules. The species that closely resembles, *Delicata tatouay* n. sp. is *D. speciosa*, but it can be distinguished by a robust branch from rays 2 and 3, rays 4 larger, and rays 8 longer compared to those of the new species. The new species is the only one with a tail, characterized by a terminal spine with rattlesnake tail-like transversal striations.

## Introduction

Currently, 13 species are assigned to *Delicata* Travassos, 1935, infecting the small intestine of armadillos: *Dasypus novemcinctus* Linnaeus, 1758; *Dasypus hybridus* (Desmarest, 1804)*; Cabassous unicinctus* (Linnaeus, 1758); *Euphractus sexcinctus* (Linnaeus, 1758); and the Southern anteater *Tamandua tetradactyla* (Linnaeus, 1758) (1–3), distributed in Brazil, Argentina, and Trinidad and Tobago (1, 3).

The greater naked-tailed armadillo, *Cabassous tatouay* (Desmarest, 1804), can be found inhabiting Uruguay, northeastern Argentina, eastern Paraguay, and south, central, and northeastern Brazil (4). It is the largest species of the genus, measuring approximately 48 cm (head-body length) and weighing approximately 4.8 kg (5, 6). In Brazil, it occurs in the Atlantic Forest, Cerrado, Caatinga, Pampas, and Pantanal near the transition to the Cerrado savanna (4, 7). They are solitary and insectivorous (8, 9), feeding on terrestrial ants and termites (10, 11). The species uses both forested and open areas but prefers forested habitats (5, 12).

This is a poorly known species of armadillo, with relatively few records in Brazilian museums (13). In the IUCN Red List, it is listed as the least concern (12), whereas in the Brazilian Red Book, it is considered data deficient (14). The main threats to the species are probably deforestation and fire, as well as hunting and persecution (14).

The use of carcasses of wild road-killed vertebrates for scientific purposes has provided discoveries for science (15–17). Considering that the greater naked-tailed armadillo is poorly known, the use of samples from these animals is an opportunity to contribute to scientific development, including helminthology, given that there is almost no information regarding helminths from this host (18).

During a parasitological survey in the small intestine of one *C. tatouay* road-killed on the BR040 highway in Rio de Janeiro state, Brazil, a new species of the nematode *Delicata* was collected and described herein.

## Materials and methods

One road-killed adult *C. tatouay* was collected in April 2011 on the federal BR-040 highway, 38 km, in Areal municipality, as part of the project “Caminhos da Fauna.” The project “Caminhos da Fauna” started in 2006, is still in progress, and comprises the pioneering study in the monitoring of road-killed vertebrates in the state of Rio de Janeiro. The database used in the study comes from the monitoring of the road-killed vertebrate along a 180.4 km stretch of the BR-040 (from 125.2 km in the municipality of Duque de Caxias, state of Rio de Janeiro, to 773.5 km in the municipality of Juiz de Fora, state of Minas Gerais).

Carcass collections are included in the SISBIO License Number: 30727-9. The animal carcasses used in this study meet and are in accordance with operation license No. 1187/2013 and authorization for capture, collection, and transport of biological material – Abio (first renewal and third rectifier) 514/2014.

The abdominal and thoracic cavities of the host specimen were opened, and the organs were placed separately in Petri dishes, washed in saline solution (0.9% sodium chloride), and dissected under a stereomicroscope to remove the small helminths. Collected nematodes were conserved in 70° ethanol. Ten specimens were clarified in a 50% alcohol/glycerin solution, mounted as temporary slides, and examined under a Zeiss Standard 20 light microscope. Drawings for morphologic and morphometric analyses were made with the aid of a camera lucida, and the images were obtained with a digital camera (Olympus DP-12) and a light microscope (Olympus BX-51). Transversal sections on the anterior, middle, and posterior parts of the body of males and females were made in order to study the synlophe. The nematodes were identified following Anderson et al. (19), Travassos (2), and Durette-Desset (20). The measurements are given in micrometers unless otherwise indicated. Means are followed by the range between brackets. The holotype, allotype, and paratypes were deposited in the helminthological collection of the Oswaldo Cruz Institute (CHIOC) in Rio de Janeiro.

## Results

**Figure 1:**
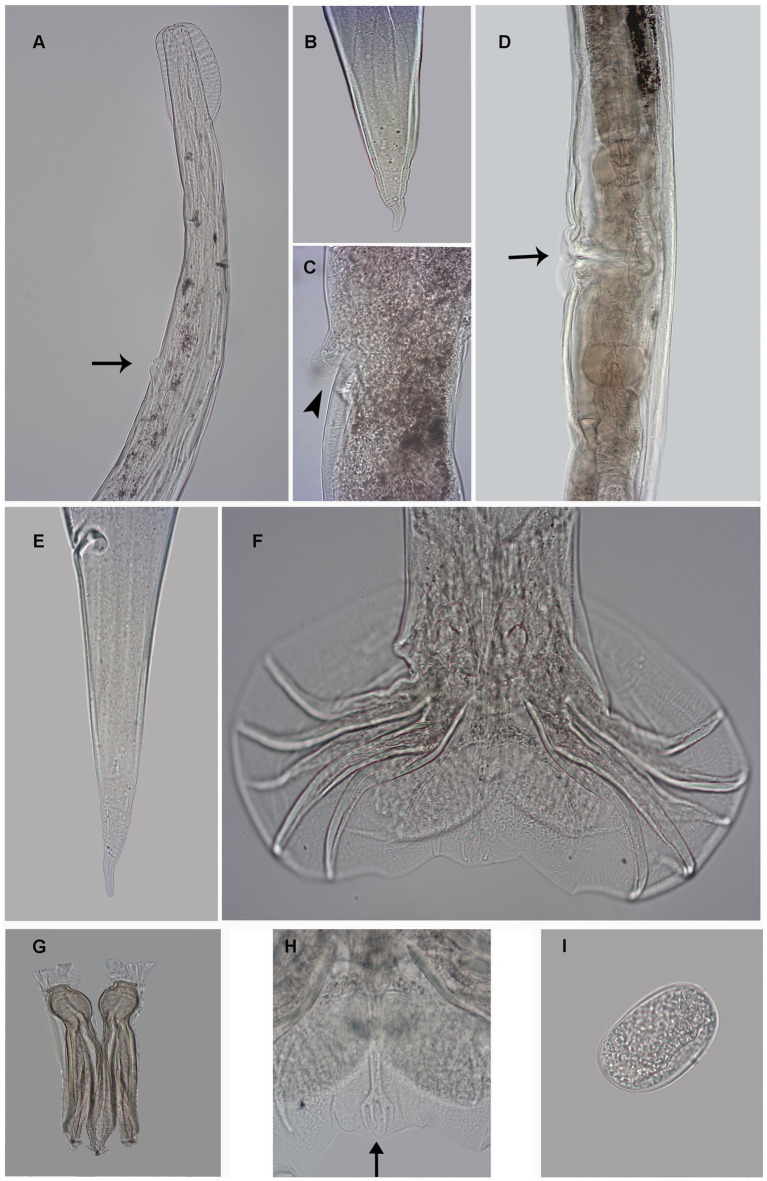
Photomicrography of female *Delicata tatouay* n. sp. **(A)** Anterior extremity, excretory pore (arrow). **(B)** Detail tail tip female. **(C)** Detail vulva (arrowhead). **(D)** Vulva (arrow). **(E)** Posterior extremity, ventro-lateral view of the anus. **(F)** Male, caudal bursa, ventral view. **(G)** Spicules. **(H)** Detail Dorsal rays (arrow) **(I)** Egg. Scale bars: **(A, D, E, F)** = 100 μm; **(B, C, G, H)** = 50 μm; **(F)** = 10 μm.

*General*: Small, slender, coiled body, with sexual dimorphism (female larger than male); presence of a cephalic vesicle. Rounded mouth opening in apical view, surrounded by two amphids, six external labial papillae. Excretory pore situated between 42 and 60% in relation to esophagus length (Figure 1A). Deirids situated anterior to excretory pore.

**Figure 2:**
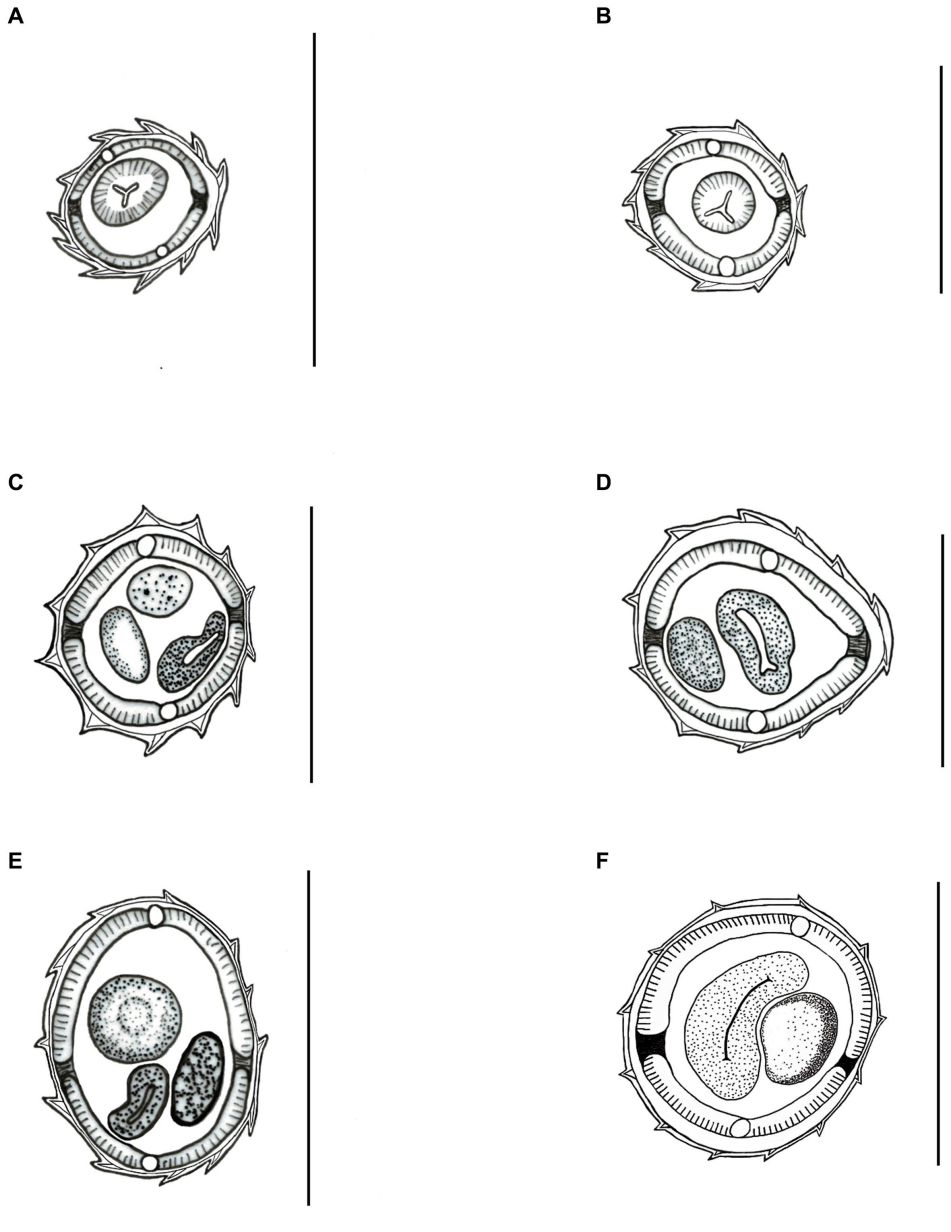
Light microscopy drawing of synlophe in transverse sections of the body from *Delicata tatouay* n. sp. **(A–C)** Female. **(A)** At the oesophago-intestinal junction; **(B)** at mid-body; **(C)** at level of the anus. **(D–F)** Male. **(D)** At the oesophago-intestinal junction; **(E)** at the mid-body; **(F)** at the level of the near caudal bursa. Scale bars: **(A–F)** = 50 μm.

*Synlophe* (studied in one male and one female): ridges appear longitudinally along the body, beginning posterior to the cephalic vesicle in both sexes. It is not observed at the proximal region of the caudal bursa in males and at the posterior extremity in females. Synlophe with 11 ridges in females and 12 in males at the level of the esophagus (Figures 2A,D); 12 ridges at mid-body in both sexes (Figures 2B,E); 12 ridges anterior to the anus in females and anterior to the caudal bursa in males (Figures 2C,F). Ridges at mid-body are slightly unequal in size in both males and females, with smaller ridges oriented from the ventral right axis and to the ventral left and from the dorsal right quadrant to the dorsal left.

**Figure 3:**
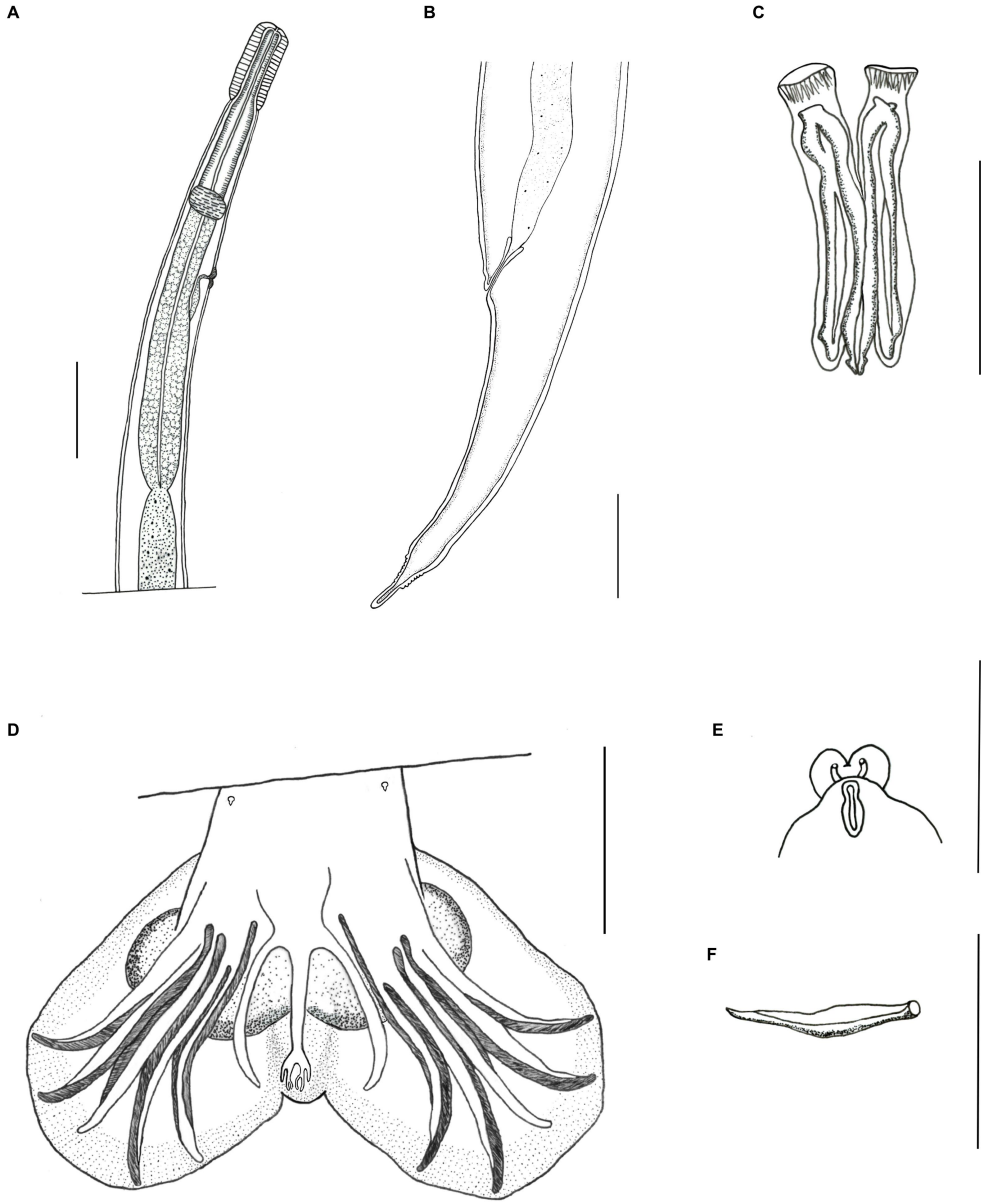
Light microscopy drawing of *Delicata tatouay* n. sp. **(A)** Female, anterior extremity, lateral view. **(B)** Female, lateral view, posterior extremity. **(C)** Male, spicules. **(D)** Male, caudal bursa, ventral view. **(E)** Male, genital cone. **(F)** Male, gubernaculum. Scale bars: **(A, B, D)** = 100 μm; **(C, E, F)** = 50 μm.

*Male* (based on one holotype and nine paratypes): length 5.97 mm (5.31–6.94 mm) and width 81 (72–107); cephalic vesicle 81 (73–83) long and 31 (30–39) wide; nerve ring, deirids, and excretory pore 212 (187–241), 234 (207–268), and 276 (253–293) from the apex; esophagus 500 (425–687) long; presence of prebursal ray 1 slightly pedunculated. Trilobate caudal bursa, right lobe slightly longer than left (Figures 1F, 3D). Rays 2 and 3 bifurcated at the second third of the trunk, with distal extremities almost reaching the bursal margin and directed ventrally. Rays 4, 5, and 6 emerging together at the base of the trunk. Ray 4 is smaller than other rays and bifurcate at the second third of the trunk. Rays 5 longer reach the bursal margin. Rays 5 and 6 bifurcate at the middle of the trunk, both distal extremities directed dorsally. All lateral rays present cuticular ornamentation. Patterns of the caudal bursa 2-1-2. Ray 8 emerging at the first third of the dorsal trunk, extending the level of the distal end of the dorsal ray but not reaching the bursa edge. Dorsal ray bifurcates at the distal extremity into 2 branches, ray 9 arising first, rays 10 divided into two branches (Figure 1H). Genital cone well developed with two membrane projections presenting papillae 7 in each extremity (Figure 3E). Papillae zero not observed. Spicules ornamented and complex in shape, wrapped in a thin sheath. Spicules are divided into two processes at the first third and show a lanceolate shape at the distal part. Spicules slightly dissimilar, left spicule 134 (126–146) and right spicule 144 (134–160) long (Figures 1G, 3C). Gubernaculum present 36 (29–45) long and 16 (12–19) wide (Figure 3F).

*Female* (based in one holotype and nine paratypes): length 7.73 mm (6.81–9.40) and width at middle body 91 (70–121); cephalic vesicle 80 (63–89) long and 37 (31–41) wide; nerve ring, deirids, and excretory pore 215 (190–239), 225 (217–316), and 286 (250–370), and from the apex, respectively (Figures 1A, 3A); esophagus length 534 (531–704); Amphidelphic, vulva situated at 1.480 (1.318–1.703) from caudal extremity with expansion digitiform (Figures 1C,D), vagina vera 44 (33–54). Anterior branch of ovejector with vestibule 62 (52–75), sphincter 40 (33–57) long and 48 (39–51) wide, infundibulum 136 (81–163) long, and uterus 1.253 (604–1.744) filled with 28 (0–61) eggs. Posterior branch of ovejector with vestibule 64 (53–78), sphincter 40 (33–54) long and 47 (39–52) wide, infundibulum 123 (76–152) long, and uterus 820 (449–1.052) with 20 (0–49) eggs. Eggs 56 (52–59) long and 33 (30–37) wide (Figure 1I). Tail 112 (107–150) long (Figures 1E, 3B). Caudal spine digitiform with fine transverse striations 20 (15–49) long (Figure 1B). Presence of phasmids 28 (43–61) from the posterior extremity.

## Taxonomic summary

*Delicata tatouay* n. sp.

*Type host*: *Cabassous tatouay*.

*Site of infection*: small intestine.

*Type locality*: Highway BR-040, Areal municipality (22°13′55.35”S, 43°7′3.93”W), State of Rio de Janeiro, Brazil.

*Deposition of type specimens:* Helminthological collection of the Oswaldo Cruz Institute in Rio de Janeiro state (CHIOC). Holotype accession number: CHIOC 39647 a; allotype accession number: CHIOC 39647 b; paratype accession numbers: CHIOC 39647 c (one male and seven females).

*Etymology:* The species epithet is due to the specific name of the host.

## Discussion

The new species belongs to the genus *Delicata,* presenting a cephalic end without a cuticular ring and a lack of cuticular plates, a female amphidelphic vulva far from the anus, a tail rounded with a caudal spine, a male with ray 5 at the same length or longer than ray 6, the presence of small post-cloacal papillae 7 at the caudal bursa, and parasites of the intestine of Xenarthra (20). This is the first species belonging to the genus *Delicata* described by *Cabassous tatouay.*

The present species is distinguished from almost all other species of *Delicata* by the longest length of the body, except for *D. khalili* and *D. appendiculata*, which show similar lengths. In addition, *Delicata tatouay* n. sp. differs from *D. soyerae*, *D. cameroni*, *D. abbai*, *D. delicata*, *D. appendiculata*, *D. uncinata*, and *D. similis* by the longest spicules (Tables 1, 2). In contrast, the species *D. ransoni*, *D. khalili*, *D.* var*iabilis*, and *D. perronae* have larger spicules than the new species.

**Table 1:** Morphometric data on male species of the genus *Delicata* in America.

Species	*Delicata khalili*	*Delicata appendiculata*	*Delicata perronae*	*Delicata soyerae*	*Delicata pseudoappendiculata*	*Delicata delicata*	*Delicata ransomi*	*Delicata uncinata*	*Delicata similis*	*Delicata* var*iabilis*	*Delicata cameroni*	*Delicata speciosa*	*Delicata abbai*	*Delicata tatouay*
Host	*Tamandua tetradactyla*	*Tamandua tetradactyla*	*Tamandua tetradactyla*	*Tamandua tetradactyla*	*Tamandua longicaudata*	*Cabassous unicinctus*	*Cabassous unicinctus*	*Cabassous unicinctus*	*Cabassous unicinctus*	*Dasypus novemcinctus*	*Dasypus hybridus*	*Dasypus novemcinctus*	*Dasypus hybridus*	*Cabassous tatouay*
Length	6.50	3.00	3.80	2.50	2.80	3.50	4.90	3.30	2.00	2.80	4.20	4.923	3.05	5.97
Width	170	77	45	41	–	78	130	70	51	62	90	65	80	80.5
Cephalic Vesicle L	50	46	62	70	–	49	56	54	40	35	32	75	38	80.5
Cephalic Vesicle W	–	–	25	21	–	–	–	–	–	–	–	–	31	33.5
Nerve ring	–	–	105	137	–	–	–	–	120	120	150	180	140	212
Deirids	–	–	124	175	–	–	–	–	–	–	–	–	155	235
Excretory Pore	–	–	140	165	–	–	–	–	160	240	200	400	190	276
Esophagus	–	380	220	200	230	360	350	320	300	290	290	315	–	500
Type	–	–	–	–	–	–	–	–	–	–	–	2-1-2	2-1-2	2-1-2
Spicule	250	115	520	105	100	99	163	81	81	180	72	222/179	56	134/143
Ratio of spicule/body total length	3.84%	3.83%	13.68%	4.20%	3.57%	2.82%	3.32%	2.45%	4.05%	6.42%	1.71%	4.50%	1.83%	2.34%
Gubernaculum L	115	69	80	58	–	63	127	–	48	29	48	139	34	35.6
Gubernaculum W	–	–	–	–	–	–	–	–	–	–	–	–	6	16
Locality	Brazil	Brazil	Brazil	Brazil	Trinidad	Brazil	Brazil	Brazil	Brazil	Brazil	Brazil	Brazil	Argentina	Brazil
Author	Travassos (21)	Travassos (21)	Durette-Desset et al. (22)	Durette-Desset et al. (22)	Cameron (23)	Travassos (24)	Travassos (24)	Travassos (25)	Travassos (25)	Travassos (25)	Travassos (25)	Lux Hoppe et al. (26)	Ezquiaga et al. (1)	Present study

**Table 2:** Morphometric data on female species of the genus *Delicata* in the Americas.

Species	*Delicata khalili*	*Delicata appendiculata*	*Delicata perronae*	*Delicata soyerae*	*Delicata pseudoappendiculata*	*Delicata delicata*	*Delicata ransomi*	*Delicata uncinata*	*Delicata similis*	*Delicata variabilis*	*Delicata cameroni*	*Delicata speciosa*	*Delicata abbai*	*Delicata tatouay*
Host	*Tamandua tetradactyla*	*Tamandua tetradactyla*	*Tamandua tetradactyla*	*Tamandua tetradactyla*	*Tamandua longicaudata*	*Cabassous unicinctus*	*Cabassous unicinctus*	*Cabassous unicinctus*	*Cabassous unicinctus*	*Dasypus novemcinctus*	*Dasypus hybridus*	*Dasypus novemcinctus*	*Dasypus hybridus*	*Cabassous tatouay*
Length	7.50	6.30	4.10	3.60	3.40	5.00	5.30	5.50	–	3.00	4.60	5.59	4.00	7.73
Width	170	110	43	45	–	87	150	90	–	67	110	95	70	90.7
Cephalic Vesicle L	90	77	62	80	–	56	78	60	–	37	43	70	45	79
Cephalic Vesicle W	–	–	21	23	–	–	–	–	–	–	–	–	30	36.5
Nerve ring	120	–	93	150	–	–	–	–	–	130	150	155	97	215
Deirids	–	–	112	185	–	–	–	–	–	–	–	–	110	225
Excretory Pore	–	–	120	172	–	–	–	140	–	210	270	337	130	286.4
Esophagus	–	500	–	230	230	370	460	340	–	290	290	–	262	533.6
Vulva	1.15	730	680	590	500	1.00	1.30	1.30	–	710	1.10	1.065	1.025	1.480
Vagina Vera	–	–	–	15	–	–	–	–	–	–	–	116	21	44
Vestibulo Ant	–	–	80	50	–	–	35	–	–	–	–	–	47	61.7
Sphincter Ant.	–	–	26	25	–	–	–	–	–	–	–	–	20	39.8
Sphincter Ant.	–	–	–	–	–	–	–	–	–	–	–	–	25	47.6
Infundibulum	–	–	35	25	–	–	–	–	–	–	–	–	40	135.8
Uterine branch	630	–	310	320	–	–	–	–	–	–	–	–	552	1.253
Vestibulo Post.	–	–	60	30	–	–	–	–	–	–	–	–	50	63.7
Sphincter Post. L	–	–	30	20	–	–	–	–	–	–	–	–	20	40
Sphincter Post. W	–	–	–	80	–	–	–	–	–	–	–	–	25	47.3
Infundibulum	–	–	32	25	–	–	–	–	–	–	–	–	40	123.3
Uterine branch	460	–	280	–	–	–	–	–	–	–	–	–	425	820
Eggs	77	69	68	63	–	63	63	70	–	–	67	58	62.6	56.5
Eggs	38	38	25	26	–	38	35	37	–	–	40	32	38.5	32.7
Tail	200	100	77	90	100	85	140	130	–	90	150	96	155	112.4
Phasmids	–	–	–	–	–	–	–	–	–	–	–	–	34	28
Caudal spine	15	–	13.5	22	–	–	–	13	–	16	18	9.6	12	20
Locality	Brazil	Brazil	Brazil	Brazil	Trinidad	Brazil	Brazil	Brazil	Brazil	Brazil	Brazil	Brazil	Argentina	Brazil
Author	Travassos (21)	Travassos (21)	Durette-Desset et al. (22)	Durette-Desset et al. (22)	Cameron (23)	Travassos (24)	Travassos (24)	Travassos (25)	Travassos (25)	Travassos (25)	Travassos (25)	Lux Hoppe et al. (26)	Ezquiaga et al. (1)	Present study

The most similar species is *D. speciosa*, but it can be differentiated because it presents a robust branch from rays 2 and 3, rays 4 larger, and rays 8 longer from those of *Delicata tatouay* n. sp. Moreover, the synlophe at the middle body of *D. speciosa* is markedly distinguished from *D. tatouay* n. sp. The first presents only four ridges situated at the ventral side and two small lateral cuticular dilatation, and the second presents 12 ridges (six ventral and six dorsal) in both sexes. Finally, *Delicata tatouay* n. sp. is the only species presenting a terminal spine in the tail with rattlesnake tail-like transversal striations.

Durette-Desset (20) characterized the synlophe of genus *Delicata* as having two lateral alae. However, Ezquiaga et al. (1) questioned this feature, suggesting not to use this character to propose a new genus once there is no synlophe of all known species, mainly *D. delicata*, that represents the type species of the genus. In addition, the known synlophe of *D. soyerae*, *D. perronae*, *D. abbai*, *D. ransomi*, and *Delicata tatouay* n. sp. have demonstrated great variability in the number of ridges. In fact, a review of the genus is required to elucidate these generic diagnostic features.

Records of the greater naked-tailed armadillo are scarce in some regions (4, 27), although it is considered globally of “least concern” (12, 28). In addition, there is almost no information about the helminth fauna of this host (18). Indeed, there is still an important lack of knowledge about parasites infecting wildlife (29), especially in highly diverse countries such as Brazil. Using road-killed vertebrates to identify new species of helminths is important not only for helminthology, but it is also essential to develop ecological research on host–parasite interaction.

## Data availability statement

The datasets presented in this study can be found in online repositories. The names of the repository/repositories and accession number(s) can be found below: http://zoobank.org/, B2C78C9B-035C-4DF6-A0B4-8D7822313471.

## Ethics statement

The animal study was approved by SISBIO License Number: 30727-9. License: No. 1187/2013 Abio: 514/2014. The study was conducted in accordance with the local legislation and institutional requirements.

## Author contributions

RO: Conceptualization, Methodology, Resources, Writing – original draft. BA: Methodology, Resources, Writing – review & editing. NO: Methodology, Resources, Writing – review & editing. CB: Methodology, Resources, Writing – review & editing. AM: Conceptualization, Methodology, Resources, Writing – original draft.
