# Supplementary material for: New species of Delicata (Molineidae: Anoplostrongylinae) parasite of Cabassous tatouay (Desmarest, 1804) from the Atlantic Forest, Rio de Janeiro, Brazil
Source: Front Vet Sci. 2024 Jan 8;10:1325263. doi: 10.3389/fvets.2023.1325263 (PMC10800746; doi:10.3389/fvets.2023.1325263)
Supplement: Supplementary file 1 [file Table_1.DOCX]

| **Specie** | ***Delicata khalili*** | ***Delicata appendiculata*** | ***Delicata perronae*** | ***Delicata soyerae*** | ***Delicata pseudoappendiculata*** | ***Delicata***  ***delicata*** | ***Delicata ransomi*** | ***Delicata uncinata*** | ***Delicata similis*** | ***Delicata variabilis*** | ***Delicata cameroni*** | ***Delicata speciosa*** | ***Delicata abbai*** | ***Delicata***  ***tatouay*** |
| --- | --- | --- | --- | --- | --- | --- | --- | --- | --- | --- | --- | --- | --- | --- |
| **Host** | ***Tamandua tetradactyla*** | ***Tamandua tetradactyla*** | ***Tamandua tetradactyla*** | ***Tamandua tetradactyla*** | ***Tamandua longicaudata*** | ***Cabassous unicinctus*** | ***Cabassous unicinctus*** | ***Cabassous unicinctus*** | ***Cabassous unicinctus*** | ***Dasypus novemcinctus*** | ***Dasypus hybridus*** | ***Dasypus novemcinctus*** | ***Dasypus hybridus*** | ***Cabassous***  ***tatouay*** |
| Length | 6.50 | 3.00 | 3.80 | 2.50 | 2.80 | 3.50 | 4.90 | 3.30 | 2.00 | 2.80 | 4.20 | 4.923 | 3.05 | 5.97 |
| Width | 170 | 77 | 45 | 41 | - | 78 | 130 | 70 | 51 | 62 | 90 | 65 | 80 | 80.5 |
| Cephalic Vesicle L | 50 | 46 | 62 | 70 | - | 49 | 56 | 54 | 40 | 35 | 32 | 75 | 38 | 80.5 |
| Cephalic Vesicle W | - | - | 25 | 21 | - | - | - | - | - | - | - | - | 31 | 33.5 |
| Nerve ring | - | - | 105 | 137 | - | - | - | - | 120 | 120 | 150 | 180 | 140 | 212 |
| Deirids | - | - | 124 | 175 | - | - | - | - | - | - | - | - | 155 | 235 |
| Excretory Pore | - | - | 140 | 165 | - | - | - | - | 160 | 240 | 200 | 400 | 190 | 276 |
| Esophagus | - | 380 | 220 | 200 | 230 | 360 | 350 | 320 | 300 | 290 | 290 | 315 | - | 500 |
| Type | - | - | - | - | - | - | - | - | - | - | - | 2-1-2 | 2-1-2 | 2-1-2 |
| Spicule | 250 | 115 | 520 | 105 | 100 | 99 | 163 | 81 | 81 | 180 | 72 | 222/179 | 56 | 134/143 |
| Ratio of spicule/  body total length | 3.84% | 3.83% | 13.68% | 4.20% | 3.57% | 2.82% | 3.32% | 2.45% | 4.05% | 6.42% | 1.71% | 4.50% | 1.83% | 2.34% |
| Gubernaculum L | 115 | 69 | 80 | 58 | - | 63 | 127 | - | 48 | 29 | 48 | 139 | 34 | 35.6 |
| Gubernaculum W | - | - | - | - | - | - | - | - | - | - | - | - | 6 | 16 |
| Locality | Brazil | Brazil | Brazil | Brazil | Trindad | Brazil | Brazil | Brazil | Brazil | Brazil | Brazil | Brazil | Argentina | Brazil |
| Author | (Travassos, 1928) | (Travassos, 1928) | Durette-Desset et al 1977 | Durette-Desset et al 1977 | Cameron, 1939 | (Travassos, 1921) | (Travassos, 1921) | Travassos, 1935 | Travassos, 1935 | Travassos, 1935 | Travassos, 1935 | Lux Hoppe  et al. 2007 | Ezquiaga et al., 2012 | Present  study |
