# Supplementary material for: New species of Delicata (Molineidae: Anoplostrongylinae) parasite of Cabassous tatouay (Desmarest, 1804) from the Atlantic Forest, Rio de Janeiro, Brazil
Source: Front Vet Sci. 2024 Jan 8;10:1325263. doi: 10.3389/fvets.2023.1325263 (PMC10800746; doi:10.3389/fvets.2023.1325263)
Supplement: Supplementary file 2 [file Table_2.DOCX]

| **Specie** | ***Delicata***  ***khalili*** | ***Delicata appendiculata*** | ***Delicata perronae*** | ***Delicata soyerae*** | ***Delicata pseudoappendiculata*** | ***Delicata***  ***delicata*** | ***Delicata ransomi*** | ***Delicata uncinata*** | ***Delicata***  ***similis*** | ***Delicata variabilis*** | ***Delicata cameroni*** | ***Delicata speciosa*** | **Delicata abbai** | ***Delicata***  ***tatouay*** |
| --- | --- | --- | --- | --- | --- | --- | --- | --- | --- | --- | --- | --- | --- | --- |
| **Host** | ***Tamandua tetradactyla*** | ***Tamandua tetradactyla*** | ***Tamandua tetradactyla*** | ***Tamandua tetradactyla*** | ***Tamandua***  ***longicaudata*** | ***Cabassous unicinctus*** | ***Cabassous unicinctus*** | ***Cabassous unicinctus*** | ***Cabassous unicinctus*** | ***Dasypus novemcinctus*** | ***Dasypus hybridus*** | ***Dasypus novemcinctus*** | ***Dasypus hybridus*** | ***Cabassous tatouay*** |
| Length | 7.50 | 6.30 | 4.10 | 3.60 | 3.40 | 5.00 | 5.30 | 5.50 | - | 3.00 | 4.60 | 5.59 | 4.00 | 7.73 |
| Width | 170 | 110 | 43 | 45 | - | 87 | 150 | 90 | - | 67 | 110 | 95 | 70 | 90.7 |
| Cephalic Vesicle L | 90 | 77 | 62 | 80 | - | 56 | 78 | 60 | - | 37 | 43 | 70 | 45 | 79 |
| Cephalic Vesicle W | - | - | 21 | 23 | - | - | - | - | - | - | - | - | 30 | 36.5 |
| Nerve ring | 120 | - | 93 | 150 | - | - | - | - | - | 130 | 150 | 155 | 97 | 215 |
| Deirids | - | - | 112 | 185 | - | - | - | - | - | - | - | - | 110 | 225 |
| Excretory Pore | - | - | 120 | 172 | - | - | - | 140 | - | 210 | 270 | 337 | 130 | 286.4 |
| Esophagus | - | 500 | - | 230 | 230 | 370 | 460 | 340 | - | 290 | 290 | - | 262 | 533.6 |
| Vulva | 1.15 | 730 | 680 | 590 | 500 | 1.00 | 1.30 | 1.30 | - | 710 | 1.10 | 1.065 | 1.025 | 1.480 |
| Vagina Vera | - | - | - | 15 | - | - | - | - | - | - | - | 116 | 21 | 44 |
| Vestibulo Ant | - | - | 80 | 50 | - | - | 35 | - | - | - | - | - | 47 | 61.7 |
| Sphincter Ant. | - | - | 26 | 25 | - | - | - | - | - | - | - | - | 20 | 39.8 |
| Sphincter Ant. | - | - | - | - | - | - | - | - | - | - | - | - | 25 | 47.6 |
| Infundibulum | - | - | 35 | 25 | - | - | - | - | - | - | - | - | 40 | 135.8 |
| Uterine branch | 630 | - | 310 | 320 | - | - | - | - | - | - | - | - | 552 | 1.253 |
| Vestibulo Post. | - | - | 60 | 30 | - | - | - | - | - | - | - | - | 50 | 63.7 |
| Sphincter Post. L | - | - | 30 | 20 | - | - | - | - | - | - | - | - | 20 | 40 |
| Sphincter Post. W | - | - | - | 80 | - | - | - | - | - | - | - | - | 25 | 47.3 |
| Infundibulum | - | - | 32 | 25 | - | - | - | - | - | - | - | - | 40 | 123.3 |
| Uterine branch | 460 | - | 280 | - | - | - | - | - | - | - | - | - | 425 | 820 |
| Eggs | 77 | 69 | 68 | 63 | - | 63 | 63 | 70 | - | - | 67 | 58 | 62.6 | 56.5 |
| Eggs | 38 | 38 | 25 | 26 | - | 38 | 35 | 37 | - | - | 40 | 32 | 38.5 | 32.7 |
| Tail | 200 | 100 | 77 | 90 | 100 | 85 | 140 | 130 | - | 90 | 150 | 96 | 155 | 112.4 |
| Phasmids | - | - | - | - | - | - | - | - | - | - | - | - | 34 | 28 |
| Caudal spine | 15 | - | 13.5 | 22 | - | - | - | 13 | - | 16 | 18 | 9.6 | 12 | 20 |
| Locality | Brazil | Brazil | Brazil | Brazil | Trindad | Brazil | Brazil | Brazil | Brazil | Brazil | Brazil | Brazil | Argentina | Brazil |
| Author | (Travassos, 1928) | (Travassos, 1928) | Durette-Desset et al 1977 | Durette-Desset et al 1977 | Cameron,  1939 | (Travassos, 1921) | (Travassos, 1921) | Travassos, 1935 | Travassos, 1935 | Travassos,  1935 | Travassos, 1935 | Lux Hoppe  et al. 2007 | Ezquiaga  et al., 2012 | Present  study |
